# *Artemisia annua* L. as a plant with potential use in the treatment of acanthamoebiasis

**DOI:** 10.1007/s00436-016-4902-z

**Published:** 2016-01-19

**Authors:** Monika Derda, Edward Hadaś, Marcin Cholewiński, Łukasz Skrzypczak, Anna Grzondziel, Agnieszka Wojtkowiak-Giera

**Affiliations:** Department of Biology and Medical Parasitology, Poznan University of Medical Sciences, 10 Fredry Street, 61-701 Poznan, Poland

**Keywords:** *Artemisia annua* L, Acanthamoebiasis, Experimental therapy

## Abstract

The treatment of acanthamoebiasis is a great problem. Most cerebral invasions end with death, and the treatment of ocular invasions is usually long-lasting and not very effective. Numerous plant extracts and substances isolated from plants, which are effective against trophozoites or cysts, have been studied in the treatment of acanthamoebiasis. However, no agents that are simultaneously effective against both developing forms of amoebae have been discovered yet. It seems that such a plant which fulfils both tasks is *Artemisia annua* L. Our studies showed that water, alcohol and chloroform extracts from the herb *A. annua* L. can be applied in general and local treatment or in combined therapy with antibiotics in the treatment of acanthamoebiasis. Extracts from this plant show not only in vitro but also in vivo effects. Studies carried out on experimental animals infected with amoebae show that the application of these extracts significantly prolongs the survival of the animals.

## Introduction

Free-living amoebae belonging to the genus *Acanthamoeba* are organisms commonly occurring in the environment surrounding humans. They feed on bacteria, mushrooms and other protozoa and are perfectly adjusted to the environment (Khan 2009). These organisms have been found in samples of soil, air, and fresh and salt water, as well as in air conditioning systems, in water supplied by waterworks, showers, sanitary appliances, swimming pools, dialysis machines and contact lens fluid. Trophozoites and cysts of amoebae have also been discovered in oceanic deposits, bottled mineral water and nasal and throat mucosal smears (De Jonckheere 1991; Mergeryan 1991; Szenasi et al. 1998; Visvesvara and Stehr-Green 1990). The first suggestions that amoebae may cause diseases in humans come from 1958 from the USA (Culbertson et al. 1959; Fowler and Carter 1965). At present, human cases of granulomatous amoebic encephalitis (GAE), aspiration pneumonia (AP) and skin inflammations, and in particular, *Acanthamoeba* keratitis (AK) are noted worldwide (Yoder et al. 2012; Marciano-Cabral et al. 2000; Marciano-Cabral and Cabral 2003; Wanachiwanawin et al. 2012; Kao et al. 2012).

Chemotherapy in the case of *Acanthamoeba* sp. infection is a great problem. Most cerebral infections end with the patient’s death, while the treatment of ocular acanthamoebiasis is usually long-lasting and not very effective. Only a few cases of effective chemotherapy in the very early stage of infection and by using highly toxic drugs have been reported (Seal 2003; Kitagawa et al. 2003; Polat and Vural 2012). In the late stage of infection, most medications are not effective (Dougherty et al. 1994; Ficker et al. 1990; Horne et al. 1994; Murdoch et al. 1998; Berra et al. 2013).

The broad applicability of chemotherapeutic agents in *Acanthamoeba* sp. infection is not doubted, but most drugs are highly toxic for humans, causing adverse reactions. Hence, alternative and natural medicinal substances which could prove suitable for use in cases of amoeba infection are sought.

Due to its potential antiparasitic properties, we decided to study the water, alcohol and chloroform extracts of *Artemesia annua*.

We investigated the possibility to use them externally as well as internally to treat infections caused by free-living amoebae, in particular in the treatment of *Acanthamoeba* keratitis (AK) and granulomatous amoebic encephalitis (GAE) or *Acanthamoeba* pneumonitis (PA).

## Material and methods

Dried aerial parts of *A. annua* L. from China were obtained from the company Magiczny Ogród (Poland). One hundred millilitre of hot distilled water was poured on the pulverized plant material in the quantity of 2–3 g and a hot water infusion (tea) was obtained (De Donno et al. 2012; Suberu et al. 2013). The next portions of the dried material in the quantity of 5–10 g were extracted with ethanol or chloroform (Sharma et al. 2014) in the Soxhlet apparatus (about 30 cycles). The obtained methanol or chloroform extracts were filtered and then vaporized to dryness under a vacuum. The dry remnant was dissolved in hot distilled water. Also, a ready-dried extract from *A. annua* 10:1 made in China (Magiczny Ogród, Poland), which was also dissolved in hot distilled water, and pure artemisinin (from the company Sigma Chemical Company) were used in the study.

The studies on the influence of the extracts on amoebae were carried out on the strain 309 *Acanthamoeba castellanii*—pathogenic for mice and isolated from the environment (Kasprzak and Mazur 1972)—and on the strain Ac32 *Acanthamoeba* sp—pathogenic for humans and isolated from a case of *Acanthamoeba* keratitis, genotype T4 (accession number KP184479). The amoebae were grown in axenic liquid cultures containing 2 % Bacto Casiton (Difco) and 10 % normal horse serum according to the procedure described by Červa (1969) and on non-nutrient agar (NN) containing 2 % non-nutrient agar Difco poured on the Petri dish and covered with a suspension of the bacterium *Enterobacter aerogenes*.

The pathogenic properties of amoebae were tested by infecting 2-week-old white mice of the BALB-c strain using the procedure described by Kasprzak and Mazur (1972) and Mazur (1984).

To the axenic culture of amoebae containing 5 × 10^4^ cells/ml, we added hot water infusion (tea), methanol or chloroform extract in the quantity corresponding to 1–300 mg of dry mass of the plant in 1 ml, ready dry extract 10:1 made in China in the quantity from 1 to 20 mg/ml and pure artemisinin (Sigma) in the quantity of 0.005 to 0.2 mg/ml. The increase in the number of amoebae was studied 24, 48 and 72 h after adding to the culture the extracts or pure substance in the log phase of growth using a Thoma counting chamber. The control was the amoeba culture without the plant extract. The half maximal inhibitory concentration (IC_50_), i.e. the lowest concentration of the studied substance inhibiting the increase of amoebae by 50 %, was determined.

The second method of investigating plant extracts was based on the amoeba grown on NN agar, on which a filter paper was saturated with the extract solution. Solutions with the concentration of 5 and 50 mg of dry mass/ml were used. The controls were filter papers saturated with sterile water. The influence of the extract on the culture increase and migration of amoebae was observed for 7 days.

Study on the influence of *A. annua* extracts on the course of infection with amoebae was tested on the mice strain BALB/c (Kasprzak and Mazur 1972; Mazur 1984). The infected mice received extracts per os, from the first day after becoming infected until the seventh day, in the volume of 0.5 ml containing 200 mg of dry mass in 1 ml.

All of the experiments were repeated five to seven times. Tests on the animals were repeated five times, using five to ten animals for each test series.

## Results

Table 1 presents the IC_50_ values for the studied extracts obtained from *A. annua*. IC_50_ was determined at 24, 48 and 72 h after infection with amoebae of the genus *Acanthamoeba*. It was found in the in vitro study that all extracts effectively inhibited the increase of amoebae and caused encystation in cultures. There was no statistically significant difference between the two studied strains of *Acanthamoeba* sp.

**Table 1:** Anti-amoebic activity of extracts from *A. annua* and pure artemisinin

Compound	IC_50_ at 24 h [mg mL^−1^]	IC_50_ at 48 h [mg mL^−1^]	IC_50_ at 72 h [mg mL^−1^]
Artemisinin pure (Sigma)	0.09*	0.12*	–
Methanol extract	15.0	12.5*	8.1*
Chloroform extract	17.4	14.1*	9.2*
Chinese water extract 10:1	26.4	21.2	15.2*
Hot water infusion (tea)	208	197	145

*P < 0.05 statistically significant difference in comparison with the control during the same time interval; *n* = 6

The pure artemisinin preparation affected amoebae from 100 to 300 times more strongly than the studied extracts. The most active anti-amoeba extract was chloroform extract. The Chinese water extract 10:1 was approximately 50 % weaker than the best chloroform extract.

**Fig. 1:**
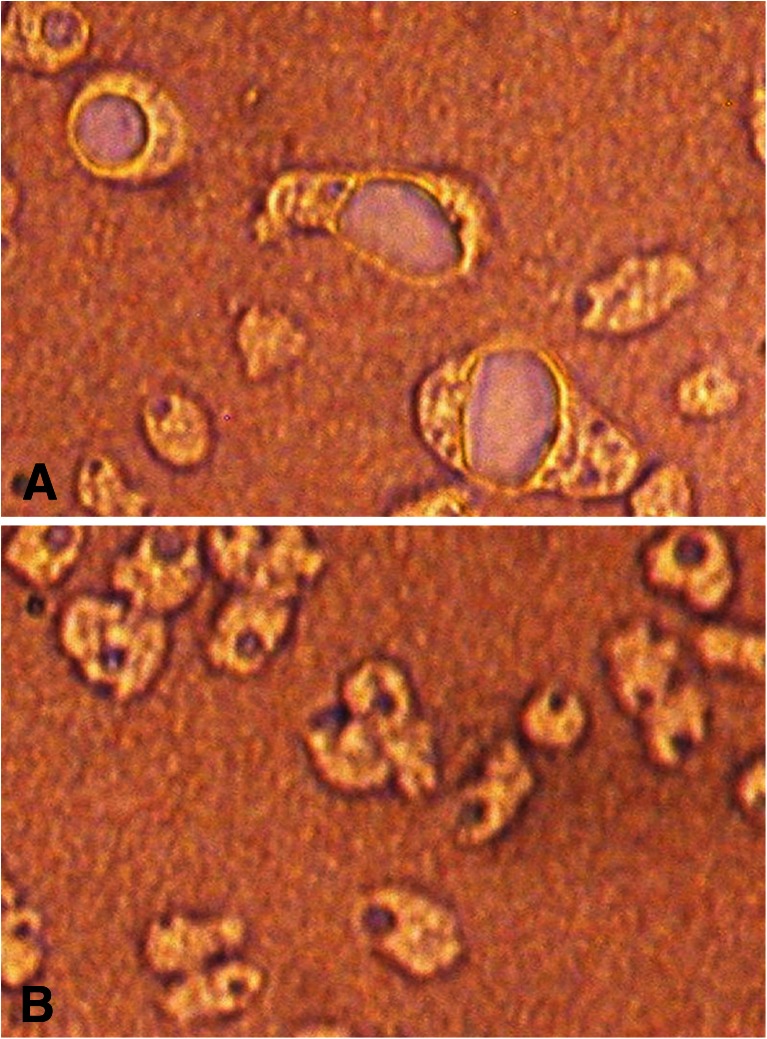
Amoebae in agar culture on a Petri dish with *A. annua* L. extract 5 mg/ml (**a**). Control culture (**b**). Magnification ×100

Studies on the effect of extracts on amoebae grown on agar plates showed that filter papers already saturated with the extract with the concentration of 5 mg/ml inhibited the growth and migration of amoebae, and moreover caused the increase in the volume of amoebae and their strong vacuolation (Fig. 1). Vacuolated amoebae did not transform into cysts and after several days became decayed. In Fig. 1, strongly vacuolated amoebae within the region of extract action can be observed.

Tests concerning the therapeutic action of plant extracts on the experimental infection with *Acanthamoeba* show that, following the application of the extracts, the animals survived considerably longer (three to four times) and infection passed into a chronic condition. Table 2 presents the survival time for mice infected with *Acanthamoeba* sp. following the application of the plant extracts.

**Table 2:** The survival time for mice infected with *Acanthamoeba* sp. following the application of the plant extracts; in each of group *n* = 6

Doses of medicines	Control	5 mg/g of tissue	10 mg/g of tissue	20 mg/g of tissue
	Survival time of infected animals (days)			
Methanol extract	7 ± 2	14 ± 2	20 ± 3	>28
Chloroform extract	7 ± 2	13 ± 3	19 ± 3	>28
Chinese water extract 10:1	7 ± 2	10 ± 4	15 ± 3	21 ± 5

## Discussion

Clinical symptoms of granulomatous amoebic encephalitis in humans are primarily strong headache, neurological disorders such as hallucinations, disorientation and visual disturbances, high fever and coma (Martinez and Visvesvara 1997). Keratitis meanwhile is characterized by severe eye pain, sensitivity to light and petechial hemorrhage (Kosik-Bogacka et al. 2010; Wanachiwanawin et al. 2012; Hadaś and Derda 2013). In the lungs, amoebae develop numerous inflammatory foci with a serous exudate containing trophozoites and cysts (Vernon et al. 2005). Skin lesions are characterized by numerous ulcers of varying extent. All forms of infection are usually chronic (Paltiel et al. 2004; Galarza et al. 2009). The chronic character of amoebic invasions in human is caused by the ability of trophozoites to transform into cysts. Cysts, in turn, are resistant to most chemotherapeutic agents.

So far, in the therapy of acanthamoebiasis, the possibility to use plant extracts and substances isolated from such plants as *Buddleia cordata* (Rodríguez-Zaragoza et al., 1999), *Pterocaulon polystachyum* (Rodio et al., 2008; Sauter et al., 2011), *Arachis hypogaea*, *Curcuma longa*, *Pancratinum maritimum* (El-Sayed et al., 2012), *Eryngium planum*, *Eryngium maritimum*, *Solidago virgaurea*, *Solidago graminifolia*, *Pueraria lobata*, *Rubus chamaemorus*, *Tanacetum vulgare* (Derda et al., 2009, 2012, 2013), *Peucedanum caucasicum*, *Peucedanum palimbioides*, *Peucedanum chryseum*, *Peucedanum longibracteolatum*, *Satureja cuneifolia*, *Melissa officinalis* (Malatyali et al., 2012a, b), *Pouzolzia indica* (Roongruangchai et al., 2010), *Salvia sclarea* (Kuźma et al., 2015), *Teucrium polium*, *Teucrium chamaedrys* (Tepe et al., 2012), *Croton pallidulus*, *Croton ericoides*, *Croton isabelli* (Vunda et al., 2012) and others have been studied.

Some of these plants are commonly used in natural medicine. They show antiseptic or amoebicidal properties. Some inhibit the development of amoebae, and others cause the encystation of trophozoites. Some extracts are lethal only for trophozoites but are not effective against cysts. A desirable property of the plant extracts is a capacity for amoebostatic and amoebicidal effects against trophozoites as well as cysts. Until now, no plants that are effective in both cases have been found. It seems that such a plant which fulfils both these requirements is *A. annua* L.

*A. annua* is an annual plant which was described by Linnaeus. It grows wild in Asia (mainly Siberia, Japan, Korea and China) and in southern Europe (Cullen 1975; Wąsowicz 2004). It was introduced to Poland, Denmark, Holland, France, Italy, Lichtenstein and Austria, where it became domesticated and bred (Tutin 1976; Wąsowicz 2004). As an introduced plant, it also occurs in North America (Żukowski and Piaszyk 1971; Cullen 1975).

*A. annua* is considered to be a medicinal plant, but in the herbal literature, it is rarely mentioned (Jędrzejko et al. 1997; Wąsowicz 2004). Its medicinal properties in malaria are considered most important (De Donno et al. 2012; Ho et al. 2013). This plant is a species of particular significance in tropical countries where the danger of malaria is the greatest (Woerdenbag et al. 1994). In China, *A. annua* is officially recognized as a medicinal plant and was listed in the Pharmacopoeia (Woerdenbag et al. 1994). It is cultivated on a mass scale in India, China and Vietnam.

The main medicinal substances of *A. annua* are artemisinins, which are sesquiterpene lactones containing an unusual peroxide bridge. The action of artemisinins involves, among other things, the creation of free radicals which facilitate the fight against parasites with the result of splitting endoperoxide bonds in their structure. This peroxide is believed to be responsible for the drug’s mechanism of action.

Moreover, artemisinin derivatives are effective against viruses (Efferth et al. 2008), protozoans (e.g. *Toxoplasma gondii* (de Oliveira et al., 2009); *Trypanosoma cruzi* (Sülsen et al., 2008); and *Plasmodium falciparum* (Sülsen et al., 2011), flatworms (e.g. *Schistosoma japonicum*, *Schistosoma mansoni*, *Fasciola hepatica*, *Clonorchis sinensis* (Fathy, 2011), bacteria and mushrooms (Lopes-Lutz et al. 2008). Artemisinin and its derivatives show cytotoxic effects against cancer cells by disrupting the cell cycle, promoting apoptosis and preventing angiogenesis. The action of artemisinins also involves inhibition of Toll-like receptors (Ho et al. 2013).

Our study showed that water, alcohol and chloroform extracts of the herb *A. annum* L. can be used in acanthamoebiasis for general and local treatment or in combined therapy with antibiotics. Medicinal substances contained in this plant show not only in vitro but also in vivo effects. In the case of animals experimentally infected with amoebae of the genus *Acanthamoeba*, it significantly prolongs their survival. Plant extracts administered to experimentally infected animals have considerably lengthened their survival time in comparison with control animals that were not given any treatment. The control animals that were infected usually died after 7 days. The animals which received a plant extract monotherapy survived for a period three to four times longer or more. In the case of animals that were not infected, the therapeutic doses of drugs given did not display any toxic activity.
